# Predicting multipotency of human adult stem cells derived from various donors through deep learning

**DOI:** 10.1038/s41598-022-25423-8

**Published:** 2022-12-14

**Authors:** Hyeonji Kim, Keonhyeok Park, Jung-Min Yon, Sung Won Kim, Soo Young Lee, Iljoo Jeong, Jinah Jang, Seungchul Lee, Dong-Woo Cho

**Affiliations:** 1 Department of Mechanical Engineering, Pohang University of Science and Technology (POSTECH), Pohang, Gyeongbuk 37673 South Korea; 2 Department of Otolaryngology-Head and Neck Surgery, Seoul St. Mary’s Hospital, College of Medicine, The Catholic University of Korea, Seoul, 06591 South Korea; 3 Department of Convergence IT Engineering, POSTECH, Pohang, Gyeongbuk 37673 South Korea; 4 Institute of Convergence Science, Yonsei University, Seoul, 03722 South Korea

**Keywords:** Stem-cell biotechnology, Stem-cell research, Predictive medicine, Machine learning

## Abstract

Adult stem cell-based therapeutic approaches have great potential in regenerative medicine because of their immunoregulatory properties and multidifferentiation capacity. Nevertheless, the outcomes of stem cell‑based therapies to date have shown inconsistent efficacy owing to donor variation, thwarting the expectation of clinical effects. However, such donor dependency has been elucidated by biological consequences that current research could not predict. Here, we introduce cellular morphology-based prediction to determine the multipotency rate of human nasal turbinate stem cells (hNTSCs), aiming to predict the differentiation rate of keratocyte progenitors. We characterized the overall genes and morphologies of hNTSCs from five donors and compared stemness-related properties, including multipotency and specific lineages, using mRNA sequencing. It was demonstrated that transformation factors affecting the principal components were highly related to cell morphology. We then performed a convolutional neural network-based analysis, which enabled us to assess the multipotency level of each cell group based on their morphologies with 85.98% accuracy. Surprisingly, the trend in expression levels after ex vivo differentiation matched well with the deep learning prediction. These results suggest that AI‑assisted cellular behavioral prediction can be utilized to perform quantitative, non-invasive, single-cell, and multimarker characterizations of live stem cells for improved quality control in clinical cell therapies.

## Introduction

Cell therapy is currently a promising therapeutic approach in which viable cells are injected, grafted, or implanted into a patient to effectively treat incurable diseases. With the maturation of research, some cell-based pharmaceuticals have been commercialized or are being used for clinical translation. Most of their cell types include adult stem cells, chimeric antigen receptor (CAR)-positive T cells, and cultured primary cells obtained from autogenic or allogeneic tissues^1^. This cell-based therapy is expected to show great potential, although drawbacks have still been reported, such as infection, chronic pain, and donor dependency, leading to varied outcomes.

Adult stem cells have attractive advantages in cell transplantation therapies because they possess immunoregulatory properties and intrinsic regeneration capacity with the potential to differentiate into multiple lineages. In particular, recent clinical studies have demonstrated that bone marrow-derived mesenchymal stem cells (MSCs) are very helpful in mediating the long-term complications of chronic inflammation caused by COVID-19 infection^2,3^. Despite these practical achievements in clinical practice, there remains a bottleneck in predicting or regulating the efficacy of cellular differentiation.

It is known that their diverse differentiation abilities are determined by their cell origin. Decades of clinical and experimental evidence have shown that large variations in the response to adjacent biomolecular signals are dependent on donors^4,5^. Several previous studies have reported that the tissue source also directs the differentiation lineage of cells^6^. However, recent studies have revealed that epigenetic memory, inspired by different cellular origins of the same donor, does not contribute to functional differentiation efficiency in vivo^7^. It can be noted that such cellular functional outcomes strongly depend on the donor. These variations limit their clinical benefits. We also faced such limitations. In our previous studies, although we successfully developed 3D printed cornea using human nasal turbinate stem cells (hNTSCs)^8–11^, a uniform quality of donor variation was not markedly ensured during the manufacturing process of clinical implants. Although some physical and chemical strategies have attempted to address these inordinate effects^12,13^, they commonly rely on in vitro conditioning to lessen possible lineages.

Deep learning-based approaches have recently been applied to analyze cellular morphologies in bioengineering and cell biology^14–16^. Several studies have used deep learning to identify cell types, segment single cells from bright-field images, and enable super-resolution in fluorescence microscopy^17–20^. In particular, deep learning could classify specific cells or predict their differentiation status. For example, a deep neural network accurately predicted the differentiation of stem cells and the early onset of multipotent stem cell differentiation in images obtained using transmitted light microscopy^21–23^. Another study showed that deep learning algorithms (e.g., convolutional neural networks (CNNs)) have been beneficial in the automatic classification and recognition of human-induced multipotent stem cell regions^24^. These studies highlight the potential for deep learning to be used in the field of cell therapy, and we believe that deep learning might be used to assess the biological features of stem cells, which have similar characteristics before differentiation. Deep learning approaches that can predict cell differentiation are expected to be widely used in the cell therapy industry.

**Figure 1:**
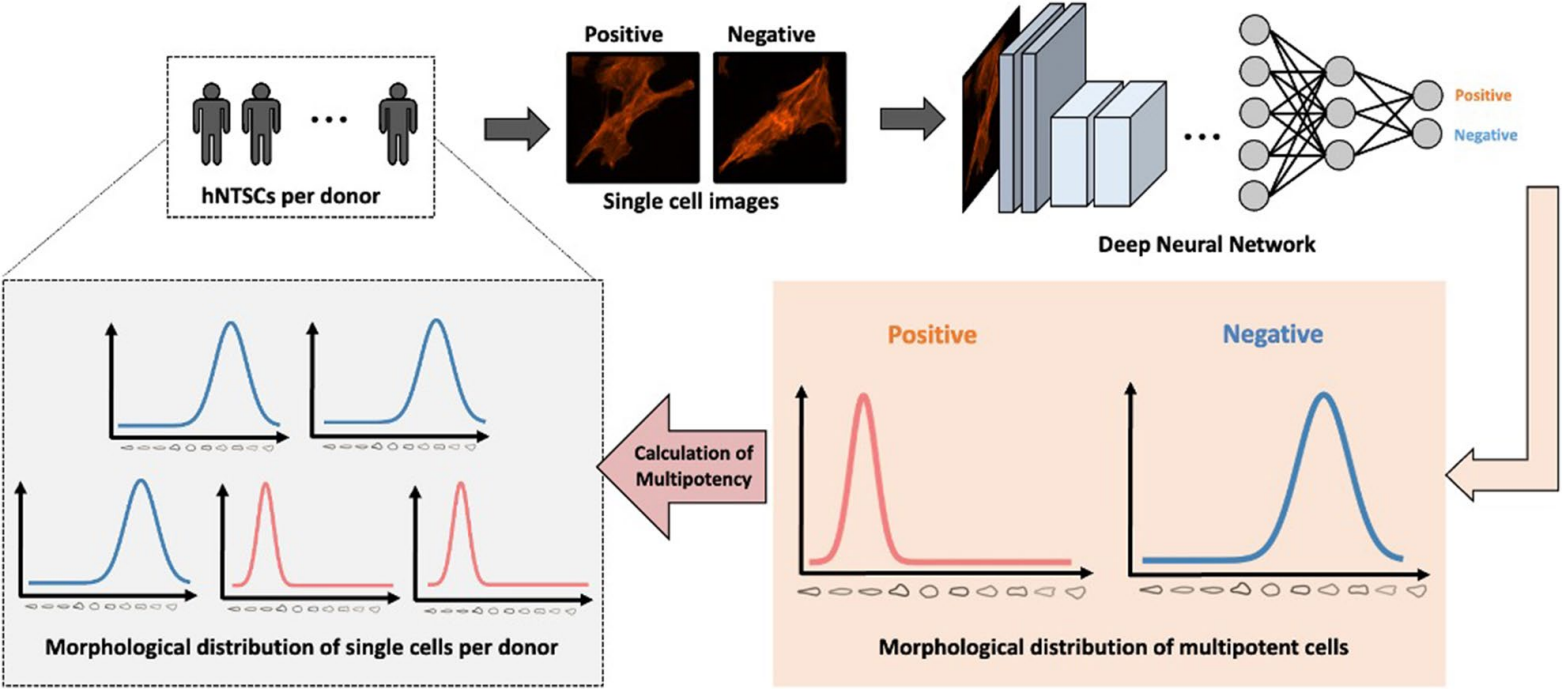
Schematic illustration of morphology-based prediction.

In this study, we employed an image-based prediction method to characterize the interaction between the cellular morphology and the multipotency of stem cells (Fig. 1). This study aims to prepare a process for clinical studies of 3D printed corneas. Therefore, we must classify the cellular sources that show better differentiation capacity, leading to the best results for bioengineered corneas. First, we obtained hNTSCs from five different donors. Then, we classified the multipotent cells by positively stained images using a stage-specific embryonic antigen 3 (SSEA-3) antibody and obtained cellular morphologies. Next, we performed CNN-based analysis to quantitatively assess the multipotency of stem cells from each donor based on their morphologies. By analyzing various model architectures and training methodologies, we established our networks using transfer learning and conducted a comparative study to determine the best-performing model. Thereafter, we validated whether the morphology-based prediction matched well with the differentiation efficacy via in vitro and ex vivo assessments.

## Results and discussion

### Comparable characteristics of patient-derived mesenchymal stem cells

**Figure 2:**
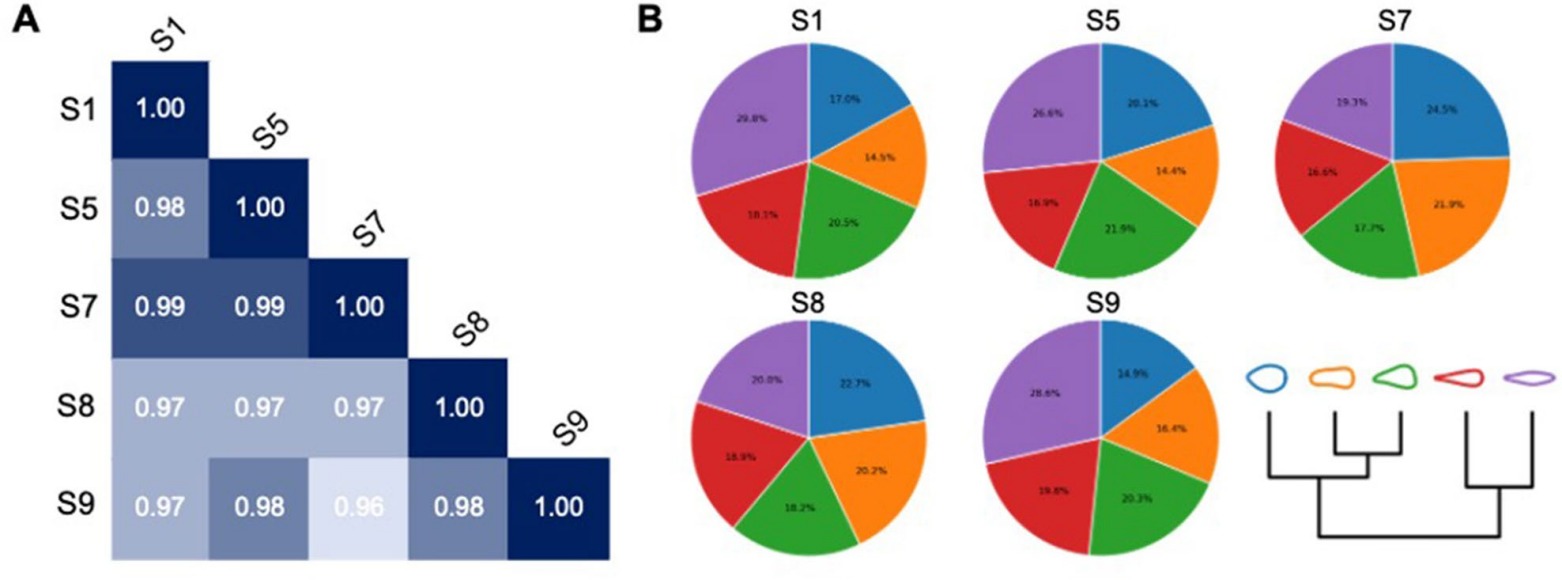
(**A**) Pearson correlation values of hNTSCs from different donors. (**B**) Morphological distribution of hNTSCs from each donor.

The process of obtaining hNTSCs from the five donors was performed at the Catholic University of Korea, St. Mary’s Hospital. This study was approved by the Internal Review Board for Human Subjects Research and Ethics Committee (KC08TISS0341), and informed consent was obtained from each donor. These cells were originally intended for transplantation into patients. Thus, all processes were performed under current good manufacturing practice (cGMP) conditions. Stem cells were labeled S1, S5, S7, S8, and S9, where the numbers represent the donor index. The proximity of hNTSCs was investigated by whole-transcriptome profiling of each group via mRNA sequencing. The level of proximity refers to Pearson’s correlation coefficient, which was computed using reads per kilobase of transcript (RPKM) of genes. Recently, Mukaka et al. presented a criterion for interpreting the size of the correlation coefficient^25^. All values of Pearson's correlation were over 0.96 (Fig. 2A), which could be interpreted to mean that all hNTSCs from different donors were correlated at a very high positive level.

We then examined the cellular morphologies based on the VAMPIRE algorithm^26^. The morphologies of the hNTSCs were confined along the contours and categorized into five cluster shapes. Although the results revealed that round (higher circularities and lower aspect ratio; average circularities: 0.469; average aspect ratio: 1.528) or uniaxially elongated (lower circularities and higher aspect ratio; average circularities: 0.262; average aspect ratio: 4.162) shapes constituted a greater distribution than the others, there were no significant differences among the representative shapes (Fig. 2B). These trends were sustained in a comparison of donors. All hNTSCs groups had diverse morphologies, but rarely showed a higher proportion of specific shapes (Fig. 2B). The above results indicate that each cell group showed similarities in gene expression and cellular morphology. However, in this study, we aim to demonstrate that multipotency differences can be predicted by cellular morphologies via in-depth gene analysis and deep learning.

### Different differentiation potency of patient-derived mesenchymal stem cells

Recent studies have reported that the efficacy of stem cell-based therapy is limited because of a lack of understanding of multipotency-related issues^27^. Most previous studies have demonstrated that the transplantation of multipotent stem cells could be an excellent candidate to regenerate malfunctioning tissues based on the multipotency itself rather than their multipotent levels^28–30^.

**Figure 3:**
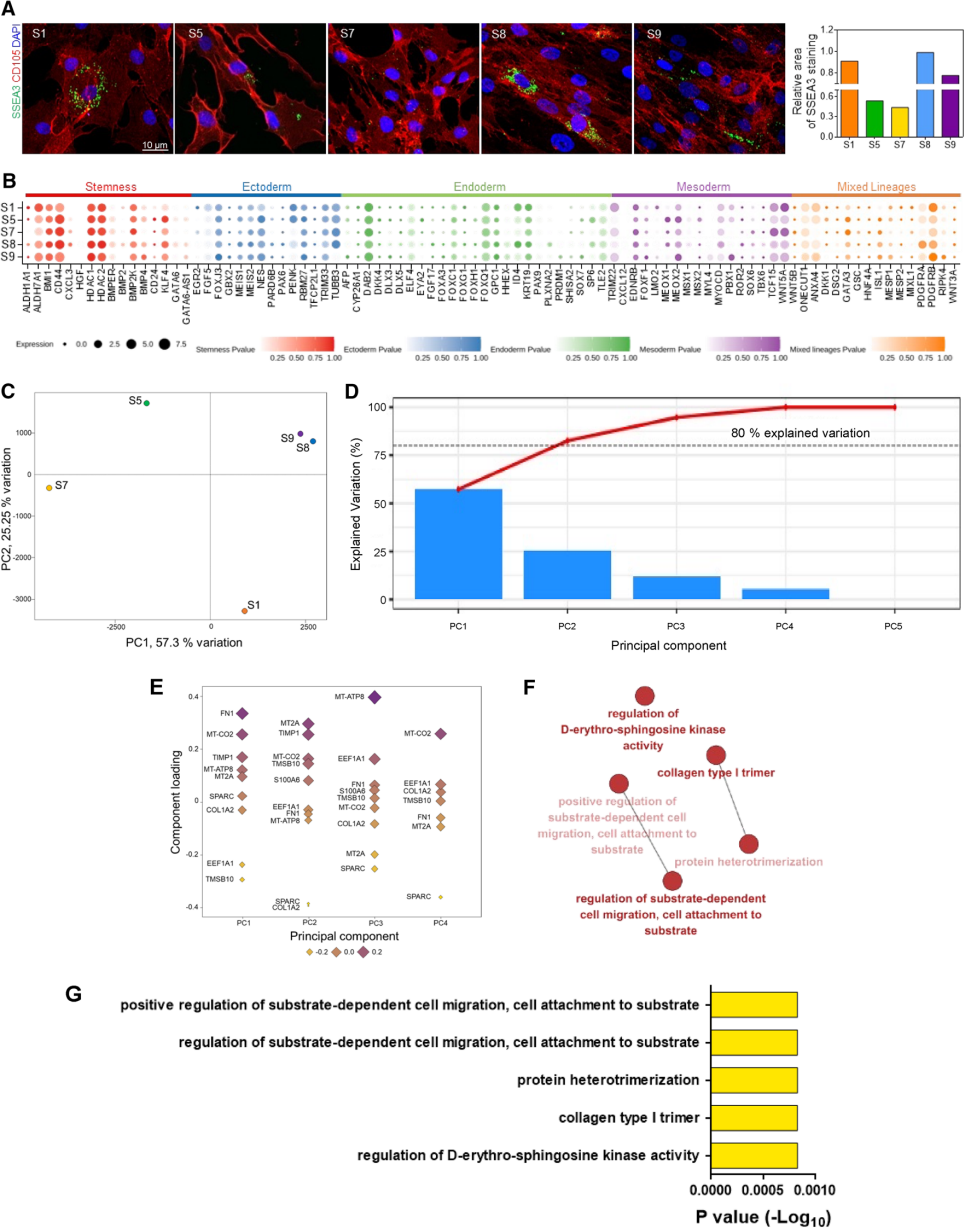
(**A**) Immunofluorescence images presenting a difference in the multipotency of hNTSCs from different donors. (**B**) Dot plot presenting expression of stemness-related genes within five different cells: the frequency of cells within a cluster expressing the gene of interest (dot size) and the level of expression of the gene (the degree of color intensity) are visualized. (**C**) Principal component analysis (PCA) and (**D**) scree plots for PCA. (**E**) PCA loading plot presenting the transformation factors of PCA. (**F**) Functional enrichment analysis visualized by each gene ontology (GO) term in which each node is linked according to a kappa score of ≥ 0.4. FDR.adj.p-value (FDR adjusted p-value). (**G**) Statistical significance and number of each GO term are presented via functional enrichment analysis. P-values were adjusted with FDR.

The researchers believe stem cells from the same organ source show similar multipotency and cellular features and expect distinguished regenerative results from their multipotency. As shown in Fig. 2, all cells from different donors showed similar genetic features and shapes. However, a difference in multipotency was revealed by the immunofluorescence staining of SSEA3 (Fig. 3A). To investigate and compare the degree of multipotency, we characterized gene expression variations in cells from different donors. A list of genes that play functional roles in stem cell maintenance^31^ was utilized. Although they expressed stemness markers evenly, the expression levels related to specific lineages were different in each donor (Fig. 3B). To further validate this finding, we conducted principal component analysis (PCA) using gene expression data and a PCA function named PCAtools^32^ with the parameter removeVar = 0.1. The results indicated that only hNTSCs from patients S8 and S9 were positioned together, and the rest departed from each other, confirming that each hNTSCs has a different cellular behavior (Fig. 3C).

To obtain details on the different gene expression patterns between each donor group, the characteristic components affecting PCA were examined. The first PCA (PC1) transformation factors accounted for approximately 89.8% of the overall volatility (Fig. 3D), and classified that hNTSCs from donors S8 and S9 have similar characteristics. Based on the PC1 gene sets (Fig. 3E), we performed functional enrichment analysis to classify their functions. PC1 genes were analyzed according to the gene ontology (GO) terms of biological processes and cellular components, and some were grouped according to their relevance. Interestingly, “positive regulation of substrate-dependent cell migration, cell attachment to substrate” (GO:1904237) and its upper GO term, “regulation of substrate-dependent cell migration, cell attachment to substrate” (GO:1904235), were observed (Fig. 3F,G). These terms were highly associated with FN1, which had the greatest positive effect on PC1 (Fig. 3E). In addition, these gene sets and GO terms were related to cellular morphologies and migration^33^. Altogether, these results imply that cellular morphologies are the most significant differences between each group, and indirectly indicate the potential of morphology-based prediction using deep learning.

### Robust prediction of multipotency based on cellular morphome

**Figure 4:**
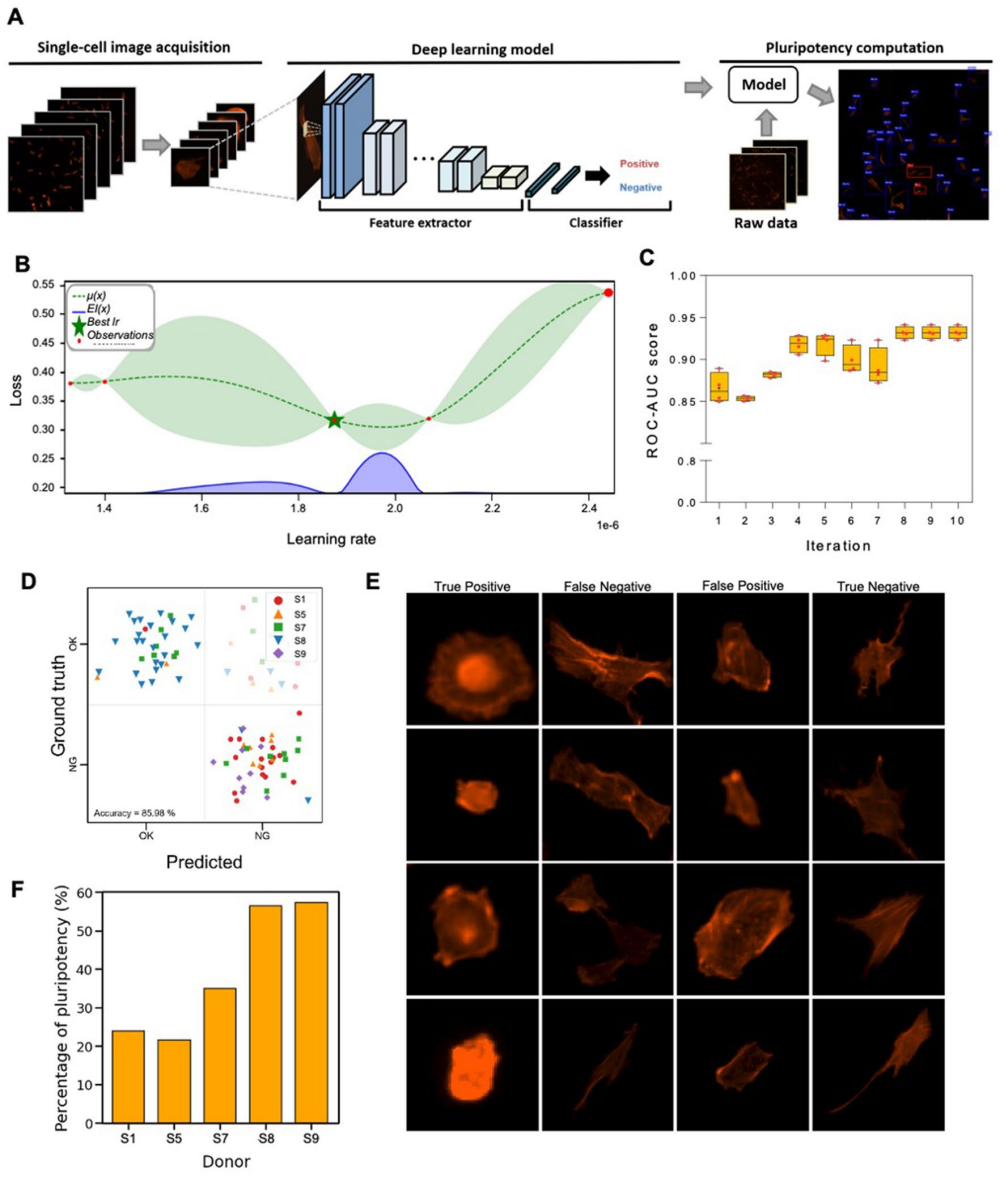
Deep learning-based multipotency prediction for hNTSCs from different donors. (**A**) Schematic images presenting the procedure of the proposed deep learning algorithm: single-cell image acquisition (preprocessing), deep learning model, and multipotency computation. (**B**) Bayesian optimization for learning rate: the results are visualized as a probabilistic model of the loss function (dotted line), uncertainty at each point (green region), and expected improvement (EI) for expensive optimization problems (blue region). (**C**) ROC-AUC score plotted over 10 iterations according to Bayesian optimization. (**D**) Confusion matrix: Predicted (x-axis) represents results through the deep learning; ground truth (y-axis) represents multipotency data revealed by immunofluorescence staining of SSEA3, and deep learning distinguishes cells with high/low multipotency. (**E**) Classification results of the proposed CNN model. (**F**) Ratio corresponding to the multipotent stem cell for each donor.

Next, we conducted experiments using a CNN model to predict the multipotency of stem cells based on their morphological information (Fig. 4A). We obtained 1,254 multipotent and 596 non-multipotent cell images and evaluated several CNN models to predict the multipotency of hNTSCs. A transfer learning-based approach was utilized as the feature extractor, with four well-performing models (VGG19^34^, InceptionV3^35^, Xception^36^, and DenseNet121^37^) pre-trained on ImageNet^38^. To improve the reliability of the results, we employed five-fold cross-validation for each model to evaluate its predictive ability by averaging the prediction results for each dataset.

We compared the prediction performances of the above models using the accuracy, sensitivity, specificity, F1 score, and AUC metrics, including the average and standard deviation values of each metric, for predicting the multipotency of hNTSCs on the testing set (Table 1). DenseNet121 achieved the best performance on all metrics except AUC, where InceptionV3 slightly outperformed it by 0.030. Therefore, we concluded that DenseNet121 was the optimal model for predicting the multipotency of hNTSCs and extracting features from immunofluorescence images of single cells.

**Table 1:** Quantitative results in comparative study of different convolutional neural network (CNN) models.

Model	Accuracy	Sensitivity	Specificity	F1	AUC
VGG19	0.826 ± 0.029	0.975 ± 0.010	0.636 ± 0.058	0.862 ± 0.021	0.874 ± 0.021
InceptionV3	0.832 ± 0.016	0.987 ± 0.014	0.636 ± 0.036	0.868 ± 0.011	0.891 ± 0.017
Xception	0.832 ± 0.015	0.986 ± 0.012	0.636 ± 0.024	0.868 ± 0.011	0.844 ± 0.010
DenseNet121	0.838 ± 0.016	0.991 ± 0.005	0.643 ± 0.031	0.872 ± 0.012	0.861 ± 0.020

Prior to the multipotency prediction of stem cells from different donors, the DenseNet121 model was trained as a feature extractor, and the optimized value of the learning rate was explored to minimize the loss function based on Bayesian optimization, which utilized prior knowledge of the optimization process and effectively identified the promising hyperparameter (Fig. 4B). To ensure robust optimization, the process was repeated five times and the results of each training iteration were merged. The validation loss gradually decreased as the number of iterations increased, and the low validation loss appeared to be convergent. The evaluation results of our optimized model were compared with those of the models learned at the learning rate used in the optimization process. We confirmed that the ROC-AUC score converged at approximately 0.93, indicating that the optimization process was robust (Fig. 4C).

To evaluate the proposed CNN model that was learned using the optimal learning rate, we assessed the confusion matrix (called the matching matrix between the ground truth and prediction) using unseen test data. The model predicted well whether hNTSCs had multipotency, and the accuracy of classification was 85.98% (Fig. 4D). Overall, deep learning predicted that round-shaped cells would express multipotency, whereas uniaxially elongated cells would not. The term “false” refers to a case where the model incorrectly predicted a class, i.e., “false negative” indicates that the predicted value is negative (non-multipotent) but the actual value is positive (multipotent) (Fig. 4D,E). The cells in the false-positive images exhibited round shapes that were more dominant, indicating multipotency. The accuracy of these misclassifications can be further improved by accumulating data from numerous cells. Nevertheless, these findings, with over 85% accuracy, indicate that the deep learning method can distinguish the multipotency of stem cells through cellular morphologies.

We then investigated the multipotency of hNTSCs from different donors using the proposed model. Cropped images of single cells from CD105-based imaging data were generated by a selective search algorithm^39^ that is widely used in the field of segmentation. Because the selective search algorithm identified differences in color and texture between the objects and combined them between adjacent similar pixels to determine the location, it was appropriate for use in our data, whose background was not noisy. The single-cell images were fed into the proposed deep learning model, and the ratio corresponding to multipotent stem cells for each donor was computed. The results showed that hNTSCs from donors S8 and S9 had higher multipotency compared to the others (Fig. 4F), which was consistent with mRNA sequencing. Based on these predictions, a downstream analysis was performed to identify the different behaviors of hNTSCs in response to the same differentiation conditions.

### Transcriptome-based evaluation of differentiated cells

**Figure 5:**
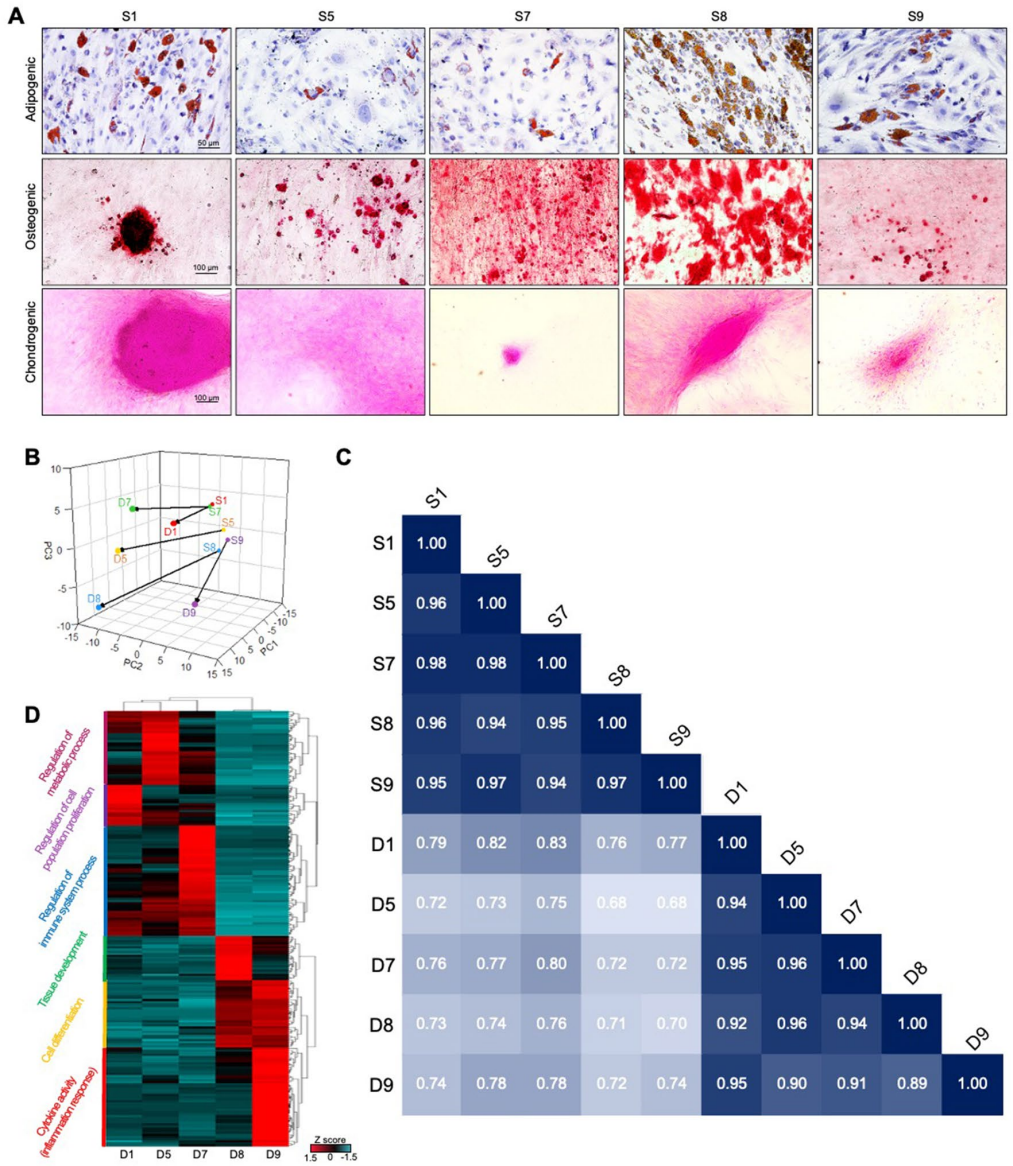
Evaluation of deep learning-based prediction by differentiating hNTSCs. (**A**) Tri-lineage differentiation results using adipogenic, osteogenic, and chondrogenic differentiation conditions. (**B**–**D**) Ex vivo differentiation results for the development of the bioengineered cornea: Comparison of original and differentiated cells visualized by (**B**) PCA plot and (**C**) Pearson correlation values. (**D**) A heat map presenting the representative functions of the differentiated cells.

To validate the morphology-based prediction, we compared the differentiation efficacy. First, we cultured hNTSCs in adipogenic, osteogenic, and chondrogenic conditioned medium. As expected, S8 exhibited the most significant differentiation results, showing a wider stained area (Fig. 5A). The remaining cells showed different results for each medium. For example, S9 showed more adipogenic cells than S7, whereas S7 displayed more osteogenic cells than S9. These characteristics can be further distinguished and predicted using deep learning technology.

Thereafter, we evaluated the differentiation efficacy via an ex vivo assessment, focusing on the development of clinically available bioengineered corneas. We encapsulated each cell in cornea-derived decellularized extracellular matrix (Co-dECM) and cultured it for 14 days at 37 °C. To assess the subsequent in-depth analysis, PCA was conducted using original stem cells (S1, S5, S7, S8, and S9) and differentiated cells (D1, D5, D7, D8, and D9). All the differentiated cells showed obviously different features compared with the original stem cells (Fig. 5B,C). Furthermore, the differentiated cells showed different features among themselves.

We also compared the gene expression patterns among differentiated hNTSCs. Despite the same differentiation conditions, the results visualized using a heat map showed different patterns depending on the donor. As shown in Fig. 5D, both D8 and D9 are related to “Cell differentiation,” which is completely identical to our prediction. The difference between D8 and D9 is that D8 is most relevant to “Tissue development” whereas D9 is more associated with “Cytokine activity.” In addition, other groups (D1, D5, and D7) are relevant to regulation-related functions, which are characteristic of hNTSCs. Altogether, these results indicate that our AI-assisted prediction based on cellular morphologies can successfully distinguish donor-dependent multipotency in that the results are practically identical to the actual differentiation results.

## Conclusion

Heretofore, the most popular considerations of stem cell therapy have focused on in vivo safety, including tissue source and cellular viability. Such processes are likely to overlook the efficacy of stem cells, resulting in negative feedback in stem cell therapy. In this study, we have successfully demonstrated the potential of an image-based prediction method for the multipotency of stem cells. The CNN-based analysis could classify multipotent cells, and the prediction matched the actual differentiation results. We will further utilize this prediction method to prepare clinical studies using bioengineered corneas. In addition, we expect that these morphology-based predictions will provide improved results for cellular treatments in clinics as well as open new avenues to related fields.

## Methods

### Cell culture and differentiation assessment

hNTSCs were obtained from the Catholic University of Korea, St. Mary's Hospital, and cultured in normal Dulbecco's modified Eagle's medium (Gibco, USA) containing 10% (v/v) fetal bovine serum (Gibco, USA) and 1% (v/v) penicillin/streptomycin (Sigma-Aldrich, USA) at 37 °C in a humidified 5% CO_2_ atmosphere. All procedures involving human subjects were approved by the Institutional Review Board of the Catholic Medical Center Clinical Research Coordinating Center (KC08TISS0341) and were conducted in accordance with the relevant guidelines and regulations. For differentiation, cells were cultured using the StemPro Adipogenesis Differentiation Kit (Gibco, USA), StemPro™ Osteogenesis Differentiation Kit (Gibco, USA), and StemPro™ Chondrogenesis Differentiation Kit (Gibco, USA), according to the manufacturer’s instructions. Adipogenic, osteogenic, and chondrogenic differentiation were evaluated using an Oil Red O staining solution (Sigma-Aldrich, USA), Alizarin Red S staining solution (Merck, USA), and Safranin O stain kit (NovaUltra, USA), respectively. For the ex vivo culture, Co-dECM was prepared as previously described. In brief, whole corneas were obtained from bovine eyeballs, washed using phosphate buffered saline (PBS) with penicillin (100 units ml^−1^) and streptomycin (0.1 mg ml^−1^). Stromal layers were separated from corneas and stirred in 20 mM ammonium hydroxide (NH4OH; 4.98 N solution in water, Sigma-Aldrich, USA) with 0.5% Triton X-100 (99.9% purity, Bio-Sesang, Korea) for 4 h. Then, the tissues were treated in hypotonic Tris hydrochloride (Tris–HCl; pH 7.4, Bio-Sesang, Korea) buffer solution for 24 h and 10 mM Tris–HCl solution with 1% (v/v) Triton X-100 for 24 h at 37 °C, resulting in Co-dECM tissues. The Co-dECM tissues were sterilized using 1% peracetic acid (32 wt% in dilute acetic acid, Sigma-Aldrich, USA) in 50% ethanol for 10 h. After decellularization, the Co-dECM was lyophilized overnight and crushed into a fine powder using liquid nitrogen and a milling machine. Co-dECM powder (0.2 g) was digested in acetic acid (10 ml, 0.5 M; Merck, USA) solution supplemented with pepsin (0.02 g; Sigma-Aldrich, USA) for 3 d to remove telopeptides in collagen molecules. After complete digestion of the Co-dECM gel (2%), the solution was filtered through a 100 μm mesh and adjusted to pH 7.0–7.4 with a solution of sodium hydroxide (NaOH, 10 M; Sigma-Aldrich, USA) on ice. Thereafter, hNTSCs at passage 6 were encapsulated in the Co-dECM hydrogel at a concentration of 5 × 10^6^ cells ml^−1^ and cultured in differentiation medium containing 10 ng ml^−1^ KGF/EGF for 14 days.

### mRNA sequencing

The libraries were prepared for 151 bp paired-end sequencing using the TruSeq stranded mRNA Sample Preparation Kit (Illumina, USA). Specifically, mRNA molecules were purified and fragmented from 1 μg of total RNA using oligo (dT) magnetic beads. The fragmented mRNAs were synthesized as single-stranded cDNAs through random hexamer priming. Double-stranded cDNA was prepared by applying this as a template for second strand synthesis. After sequential end repair, A-tailing, and adapter ligation, cDNA libraries were amplified with polymerase chain reaction (PCR). The quality of the cDNA libraries was evaluated using the Agilent 2100 BioAnalyzer (Agilent, USA). They were quantified using the KAPA Library Quantification Kit (Kapa Biosystems, USA) according to the manufacturer’s library quantification protocol. Following cluster amplification of denatured templates, paired-end sequencing (2 × 151 bp) was performed using Illumina NovaSeq6000 (Illumina, USA).

### Immunofluorescence staining

The cells were fixed with paraformaldehyde solution in PBS (4% w/v), permeabilized with Triton X-100 (0.1%), treated with bovine serum albumin (Affimetrix, USA) in PBS (3%) for 1 h to block the nonspecific binding, washed in PBS thrice for 15 min, incubated with Anti-SSEA3 antibody (Abcam, UK) and Anti-CD105 antibody (BD, USA) overnight at 4 °C, washed with PBS, exposed to Alexa Fluor 488 goat anti-rat antibody (Invitrogen Life Technologies, USA) for 1 h at 37 °C, and counterstained with 4',6-diamidino-2-phenylindole (DAPI). Images of the stained cells were obtained using an FV1000 Olympus confocal microscope (Olympus, Japan).

### Analysis of morphological distribution of hNTSCs per donor

First, we segmented the images of the cells using CellProfiler (see https://cellprofiler.org/ for more information). We used Visually Aided Morpho-Phenotyping Image Recognition (VAMPIRE) software (https://github.com/kukionfr/VAMPIRE_open), an unsupervised machine learning algorithm that quantitatively analyzes the morphology of segmented images of cells and finds the correlation between the shape modes in a dendrogram. VAMPIRE extracted the equidistant points along the contour of each cell, found the eigenshape vectors through principal component analysis (PCA), and applied the K-means clustering algorithm for representative shape modes. The number of coordinates used to extract the contours was 50 and the number of shape modes was 20.

### Dataset preprocessing for training

We preprocessed the hNTSCs dataset before feeding the stem cell images into our proposed deep learning model. To focus on single cells only, we used the images of cells stained with CD105 using a confocal microscope and manually cropped all regions except the cell region. Images of hNTSCs were used, and images with SSEA3 intensities higher than 90 were labeled as multipotent cells (Positive), whereas images with an intensity less than 90 were labeled as non-multipotent cells (Negative). We obtained 1,254 multipotent and 596 non-multipotent cell images and evaluated several CNN models to predict the multipotency of hNTSCs. We resized the cell images to 224 × 224 for the input dimensions of the model and applied min–max normalization to the images of the stem cell. Furthermore, our cell image dataset (1,850 images) was split into training (1,295 images; 70%), validation (185 images; 10%), and testing (370 images; 20%) data to optimize and evaluate the deep learning models. We also augmented the training images by rotating them 90° and flipping them horizontally and vertically.

### Deep learning

We utilized a binary classification model to predict whether hNTSCs had multipotency using Keras libraries. In the transfer learning-based approach, the proposed model consists of two parts: a feature extractor and a classifier. As a feature extractor (denoted as the base model), we loaded the DenseNet121 model and excluded the ImageNet classifier from the top layers. The entire network was trained without freezing the layers. Although freezing all layers of the pre-trained model has the clear advantage of allowing for a faster training process, we used a fine-tuning strategy, mainly because our single-cell dataset differs from the ImageNet dataset. The top classifier had a dropout layer with a rate of 0.4 (the same rate as below); a 2D global max-pooling layer for spatial data; a dropout layer; a hidden layer (64 units) with ReLU as a nonlinear activation function; a batch normalization layer; a dropout layer; and a final output layer of two units (binary output) with the softmax activation function. The Adam optimizer was used for model optimization along with a categorical cross-entropy loss function. The proposed model was trained for 50 epochs using the early stopping callback method with 10 patients (number of epochs with no improvement in validation loss).

## Data Availability

The datasets generated and analyzed during the current study are available in the Gene Expression Omnibus (GEO) repository with accession number GSE210678.
